# Social Media Influencer Marketing as a Clinical Trial Recruitment Modality: Tutorial Informed by One Study’s Approach

**DOI:** 10.2196/92813

**Published:** 2026-08-11

**Authors:** Timothy B Plante, Jingyi Cao, Tamunotonye Harry, Yuanyuan Feng, Azuka Amaka Ngige, Hailey N Miller, Kayla Ferro, Marian E B Budu, Stephen P Juraschek

**Affiliations:** 1 Department of Medicine, Larner College of Medicine, University of Vermont, 360 S Park Dr, Suite 206B, Colchester, VT, 05401, United States, 1 802 656 3688, 1 802 656 8965; 2 Department of Medicine, Beth Israel Deaconess Medical Center, Boston, MA, United States; 3 Department of Computer Science, College of Engineering and Mathematical Sciences, University of Vermont, Burlington, VT, United States; 4 Division of Cardiology, School of Medicine, Johns Hopkins University, Baltimore, MD, United States; 5 School of Nursing, Johns Hopkins University, Baltimore, MD, United States

**Keywords:** clinical trials, recruitment, social media, influencers, marketing, Dietary Approaches to Stop Hypertension, hypertension

## Abstract

**Background:**

Influencer marketing (paid promotion by individuals with large, engaged social media followings) has become a major commercial advertising strategy, projected to reach US $32 billion globally in 2025. Clinical trials increasingly recruit through digital channels such as social media advertisements and patient portal messages. However, to our knowledge, influencer marketing has not been described as a clinical trial recruitment modality, and no practical guidance exists for investigators who wish to use it.

**Objective:**

We provide a step-by-step tutorial describing how we developed and deployed a social media influencer recruitment video for the GoFreshSE (Groceries for Residents of Southeastern USA to Stop Hypertension) trial, a decentralized pilot randomized trial of home-delivered Dietary Approaches to Stop Hypertension–pattern groceries for adults with elevated blood pressure in Florida, Georgia, and Tennessee. We also provide a reusable preparation framework for other study teams.

**Methods:**

Using Cameo Business, an online marketplace where public figures record short promotional videos, we filtered candidates by audience location (Atlanta, Miami, and Nashville), follower count (≥100,000), and price (US <$3000) and then selected an influencer on the basis of audience demographics relevant to our recruitment priorities. We transcribed the influencer’s sample videos to characterize his speaking style, drafted a study script, and used a large language model (GPT-4o) solely to adapt the script’s tone; the adapted script was reviewed by the study team and approved by the institutional review board before use. After the influencer recorded the video, we corrected gaze and adjusted pace using openly available tools, added framing and captions, and deployed the advertisement on Facebook and Instagram through Meta’s advertising platform.

**Results:**

A qualifying pool of 57 influencers met our follower and cost thresholds. The selected influencer delivered a high-quality video 4 days after booking. The total Cameo cost was US $660, including a 75-day license and service fee, and the elapsed time from script development to a live advertisement was less than 2 months. The final 47-second video was rendered to satisfy Meta and TikTok placement and aspect ratio requirements while retaining the platform-required watermark. We report each step (marketplace search, booking request, script adaptation, ethics review, video editing, and deployment) in sufficient detail to be reproduced.

**Conclusions:**

Social media influencer marketing through Cameo Business is a rapid, low-cost mechanism for producing clinical trial recruitment videos and is feasible within typical trial timelines and budgets. To our knowledge, this is the first tutorial to document the procedure end to end, and it surfaces practical, ethical, and authenticity considerations, including governance of generative artificial intelligence and the limits of permissible video editing, that investigators should weigh before adopting this modality. The comparative effectiveness of influencer-based recruitment will be evaluated separately.

## Introduction

### Background

Clinical trials provide foundational information that drives evidence-based clinical practice [1]. Recruitment is a key component of clinical trial success; however, 80% of clinical trials do not achieve their recruitment targets during their planned recruitment window and >50% never achieve their target recruitment levels [2,3]. Inadequate trial recruitment is a major driver of early trial closure [4], thereby threatening the generation of evidence to help inform clinical care. Compounding trial recruitment woes is the shift in commercial advertising platforms from legacy methods (eg, newspaper advertisements, printed mailers, and telephone calls) to digital modalities (eg, website banner advertisements, search engine advertisements, and social media advertisements) [5,6]. Clinical trials have begun to follow suit and adopt digital recruitment strategies such as social media advertisements and patient portal messaging (eg, MyChart) [7-11]. While some studies have reported improved recruitment efficiency and lower costs relative to legacy recruitment methods [12], underenrollment issues persist [13-15]. There is a need to pursue newly emerging digital advertising modalities to address recruitment woes.

Influencer marketing is an emerging advertisement modality whereby individuals with a strong online presence participate in marketing of products or services [16]. This modality is expected to garner US $32 billion in revenue in 2025, up from US $10 billion in 2020, and is widely used among commercial brands, with 64% reporting the intention to partner with influencers in 2025 [17]. Influencers have been found to drive specific negative health behaviors among children and young adults, such as initiation of smoking, marijuana use, and consumption of calorie-dense foods [18,19]. Influencers may therefore also be effective at promoting other health-related behaviors, including clinical trial participation [18]. Despite the promise of influencer marketing, we are unaware of any reported use of influencers for clinical trial recruitment. One major barrier to the uptake of influencer-based recruitment may be the lack of any description of its implementation. We therefore provide this tutorial based on our experience implementing influencer marketing for recruitment in a clinical trial. A future manuscript will describe the efficacy of this modality for recruitment.

### Objective

This study aimed to describe the development of an influencer marketing campaign for a hypertension-focused clinical trial and provide a preparation framework for persons looking to replicate this work (Table S1 in Multimedia Appendix 1).

## Methods

### Groceries for Residents of Southeastern USA to Stop Hypertension Trial Design

The GoFreshSE (Groceries for Residents of Southeastern USA to Stop Hypertension) trial (ClinicalTrials.gov ID: NCT06891911) is an ongoing study sponsored as part of the American Heart Association (AHA) Health Care by Food initiative that is designed to evaluate the effect of remotely delivered Dietary Approaches to Stop Hypertension (DASH) diet-compliant groceries on blood pressure [20]. Key inclusion criteria include (1) adults aged 18 years or older who (2) live in Florida, Georgia, or Tennessee, and (3) have elevated blood pressure (defined as systolic blood pressure between 120 and 160 mm Hg and diastolic blood pressure <110 mm Hg). Principal exclusion criteria include severe diabetes, active cardiovascular disease, cancer diagnosis or treatment in the last 2 years, active inflammatory bowel disease, pregnancy, or significant food intolerances and dietary requirements. Target enrollment is at least 150 adults.

This study builds off of the GoFresh (Groceries for Black Residents of Boston to Stop Hypertension Among Adults Without Treated Hypertension; ClinicalTrials.gov ID: NCT05121337) and GoFreshRx (Groceries for Black Residents of Boston to Stop Hypertension Among Adults With Treated Hypertension; ClinicalTrials.gov ID: NCT05393232) trials [21-23], which tested home-delivered groceries consistent with the DASH diet versus grocery stipend among Black adults in food deserts in Boston, Massachusetts. Participants in GoFresh and GoFreshRx had study visits in research clinics in Boston, Massachusetts. In contrast, GoFreshSE is implementing the decentralized clinical trial model [24], recruiting adults in Nashville, Tennessee; Atlanta, Georgia; and nonpanhandle Florida communities and sending trained clinical examiners (GetLabs Inc) to the participant’s home to carry out study activities. As part of the GoFreshSE trial, participants are randomized to receive either DASH-pattern groceries delivered to their home for 4 weeks or a US $300 grocery stipend. All participants receive blood pressure monitors for use during the study. GoFreshSE uses multimodal recruitment techniques, including conventional still image advertisements on Meta platforms (ie, Facebook and Instagram), Epic MyChart patient portal messages, and community-based recruitment.

### GoFreshSE Recruitment Website

A GoFreshSE recruitment website was developed to aid in online recruitment and monitoring the efficacy of digital recruitment methodologies, including video advertisements. A commercial website development platform (Weebly, Block, Inc) with responsive design format was used to ensure correct website rendering on mobile devices and computers. Each page includes a footer listing the sponsor name, the institution of the principal investigator (PI), study contact information, the PI’s name, and institutional review board (IRB) number. The website is separated into several pages. The home page includes details about the GoFreshSE trial, the study phone number and email, and hosts an embedded REDCap (Research Electronic Data Capture) secure form that can be used by website visitors to undergo trial prescreening [25]. An “about” page includes additional details about the DASH diet plan, additional inclusion criteria, funding information, and ClinicalTrials.gov registration information. A “meet the team” page includes photographs and brief biographies about members on the study team. A “contact” page includes phone numbers to reach the study, the contact dietitian, and the physical address of the study team. Website visitation is monitored using a self-hosted instance of the open-source, privacy-focused web analytics platform, Matomo. This platform captures common website visitation data including date or time of visitation, pages visited, and referral source. The domain www.gofreshsoutheast.org was purchased and used as the primary domain for the GoFreshSE trial. The domain www.gofreshstudy.com was also purchased and was set to redirect to the primary domain.

### Influencer Marketing Advertisement Development Using Cameo and Cameo Business

Cameo (Barron App, Inc) is an online service where users can hire famous individuals (eg, persons with large followings on social media, actors, athletes, etc; hereafter “influencers”) to record custom short-form videos for personal use, such as for gifts for friends or family. Cameo Business includes a subset of these influencers who will record short-form videos for companies to be used in influencer marketing. Such influencers are given up to 7 days to record a video after a request is made. Influencers may choose to decline the video request. Rerecordings are allowed at Cameo’s discretion if the influencer’s recording quality was poor, if the licensee’s name was said incorrectly, or if the recorded content deviated from the request of the licensee [26]. Use of the influencer marketing video is specified by the Cameo Business terms of service as is described in Textbox 1.

The base license allows 15 days of use and may be extended to a total of 180 days. It is also possible to extend the license beyond 180 days (up to a total of 360 days) with influencer approval. Each 15-day extension is 25% of the base license price. For example, if the influencer’s fee is US $1000 for a video, extending it an additional 30 days beyond the initial 15 days would cost an additional US $500. Under the same example base fee, a full 180-day license would extend the original license by 165 days and would cost an additional US $2750. Cameo also charges a service fee, which is not explicitly defined in their terms of service but in our experience, this fee was 10% of the total cost of our order.

Textbox 1. **Cameo Business terms of service (adapted). The terms of service presented here were abbreviated and simplified by the authors. The full terms of service are available on the Cameo Business website [27].**

- The video is not owned by the recipient but instead comes with a temporary license for its use.
- After receiving the video, the recipient has up to 60 days to edit, adapt, or prepare the video for use. There cannot be misrepresentation of what the influencer said or the substantive message of the original video. The Cameo watermarks must be visible and unedited. The recipient must have rights to use any added music or images.
- After the video is prepared, the license period begins. During the license period, the video may be used on the recipient’s social media accounts, website, emails, apps, or run as an online advertisement on digital platforms or advertisement networks. It cannot be used on television, on streaming platforms (eg, Netflix), or on billboards or other outdoor advertisements.
- After the license expires, any organic posts on social media do not need to be removed (eg, if the video is posted on the social media feed of the recipient’s social media page, the old post does not need to be removed), but it no longer may be posted as an advertisement anywhere.

### Cameo Business Marketplace

As of May 22, 2025, the Cameo Business marketplace allowed users to search among 872 influencers. Because talent joins and leaves the marketplace, this count changes over time; the cited figure reflects the marketplace on the date searched [28]. Influencers can be searched by personal or audience attributes. Personal attributes include the following:

1. Industry for which they are known.
2. Cost per video.
3. Number of followers on social media.
4. Sex.

Audience attributes include the following:

1. Sex: female or male; determined by ≥40% of their audience being of that type.
2. Age: 18-24 years, 25‐34 years, 35‐44 years, or 45‐54 years; determined by ≥20% of their audience being of an age band. There are no age bands above 54 years.
  - Note: the 13‐17 years age band following is reported in each influencer’s profile, but this age band cannot be used as a search query.
3. Popularity by country: determined by ≥5% of their audience being located in that country.
4. Popularity by city: determined by ≥1% of their audience being located in that city.
5. Income: US $0 to US $100,000 by intervals of US $20,000. Determined by audiences at least 20% in that range.

Cameo Business does not specify how the audience attributes are determined, but the authors suspect that at least some of these details are drawn from publicly available profile data from a subset of their followers on social media.

### Our Steps to Identify Possible Influencers

We limited to influencers who were popular in Atlanta, Miami, or Nashville. After reviewing the distribution of followers and charges, we opted to exclude influencers with <100,000 followers on their largest social media account or a cost of ≥US $3000.

### Ethical Considerations

The GoFreshSE trial (ClinicalTrials.gov ID: NCT06891911), including all recruitment materials described in this tutorial, was reviewed and approved by the Beth Israel Deaconess Medical Center IRB (protocol 2025P000102). The influencer script was reviewed and approved as an amendment to that protocol. This tutorial reports the procedures used to produce and deploy a recruitment advertisement, together with publicly available marketplace data; it does not report data collected from human participants. No separate ethics review was sought for the tutorial itself. The influencers described in the Results section are public figures who list their services, prices, and audience characteristics on a public commercial marketplace; that information is not private information, and no information about them was obtained through any intervention or interaction undertaken for research purposes. The work reported here therefore does not meet the definition of human subjects research under 45 CFR 46.102(e) [29].

All GoFreshSE participants provide written informed consent before any study procedure is performed. No participant data are reported in this tutorial, so neither additional consent nor a waiver of consent was required. Neither Mr Hue Jackson nor any of the influencers summarized in the Results section is a research participant, and their inclusion in this manuscript therefore does not require research consent. Mr Jackson was engaged as a paid vendor under a commercial contract: the Cameo Business terms of service he accepted grant the licensee the right to use, edit, and publicly distribute the recording as an advertisement (described in the Results section), and the booking request disclosed that the video would be used to recruit participants for a clinical trial and would be run as a paid advertisement on social media platforms (Table S3 in Multimedia Appendix 1). The resulting advertisement was publicly deployed under his name and likeness. Information reported about the remaining influencers is limited to what each has published on a public marketplace listing: professional identity, price per video, follower counts, and platform-derived audience characteristics.

No individually identifiable participant information appears in this manuscript or in any supplementary material. The influencer data presented in the Results section were abstracted from publicly viewable Cameo Business listings and describe platform-derived, aggregate audience characteristics rather than private information about any individual. No influencer is identified in this manuscript as having been excluded from consideration on the basis of any particular selection criterion. Website visitation is monitored using a self-hosted Matomo instance, and no directly identifying information is retained. Prescreening responses submitted through the embedded REDCap form are transmitted over an encrypted connection and stored on institutionally managed, access-controlled servers. This descriptive manuscript does not include any data captured by Matomo or REDCap.

No compensation was provided to any human participant for the activities described in this tutorial, which involved no human participants. Mr Jackson received US $660 through Cameo Business (US $300 for the video and its 15-day license, US $300 for a 60-day license extension, and a US $60 service fee), against a fee he lists publicly on the marketplace. He holds no financial interest in the trial and had no role in the design, conduct, analysis, or reporting of the trial or of this tutorial.

**Figure 1.**
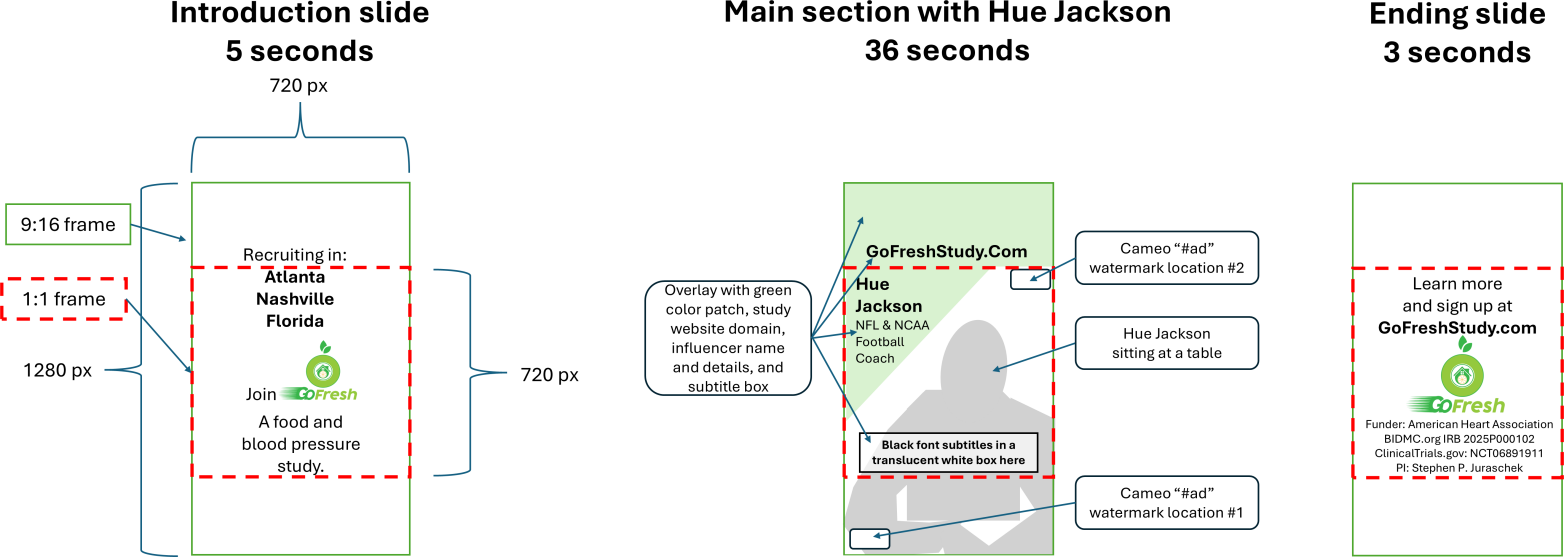
Production of the influencer recruitment video for the GoFreshSE decentralized hypertension trial; the 44-second edited video is shown before the appended 3-second Cameo slide, which together yielded the 47-second deployed video. The green rectangle represents the border for a 9:16 aspect ratio frame and the red-dashed square represents the border for a vertically centered 1:1 aspect ratio frame. These borders were not included in the video. The Cameo watermark in the main video moves between locations 1 and 2 and cannot be edited out per the Cameo Business terms of service (described in the Results section). The received video also included 3 seconds of a slide at the end that included Mr Jackson’s profile picture and name and the words “Book now on Cameo.” It was unclear from the review of the terms of agreement whether this slide was required to be included in the video, and the slide was appended to the end of the 44-second video for a total length of 47 seconds. Cameo Business representatives later clarified that this slide was not required to be included in the final video, but by this point the video was already deployed on Meta platforms, so the full 47-second video was used. The final video can be seen as a historical post on the GoFresh Facebook account’s feed, accessible at the following URL: https://www.facebook.com/61550106065304/videos/join-our-clinical-trial-to-study-a-dietary-approach-to-improve-blood-pressure/25553798757589592/. Due to license terms (described in the Results section), we were unable to post this video in digital video archival platforms.

No images of research participants appear in this manuscript or in any supplementary material, and no image permits the identification of any individual participant or user. Figure 1 is a schematic representation of the layout and timing of the deployed video and contains no photographs of any person. Mr Hue Jackson is named in the text as the public figure who recorded the recruitment video under a commercial contract; because he is neither a research participant nor a user of the intervention, and because the video in which he appears was created for, and distributed as, a public advertisement, consent for identification was neither required nor sought.

No images of research participants appear in this manuscript or in any supplementary material, and no image permits the identification of any individual participant or user. Figure 1 is a schematic representation of the layout and timing of the deployed video and contains no photographs of any person. Mr Hue Jackson is named in the text and in the Acknowledgments section as the public figure who recorded the recruitment video under a commercial contract; because he is neither a research participant nor a user of the intervention, and because the video in which he appears was created for, and distributed as, a public advertisement, consent for identification was neither required nor sought.

## Results

### Influencers Identified in the Cameo Business Marketplace

**Figure 2.**
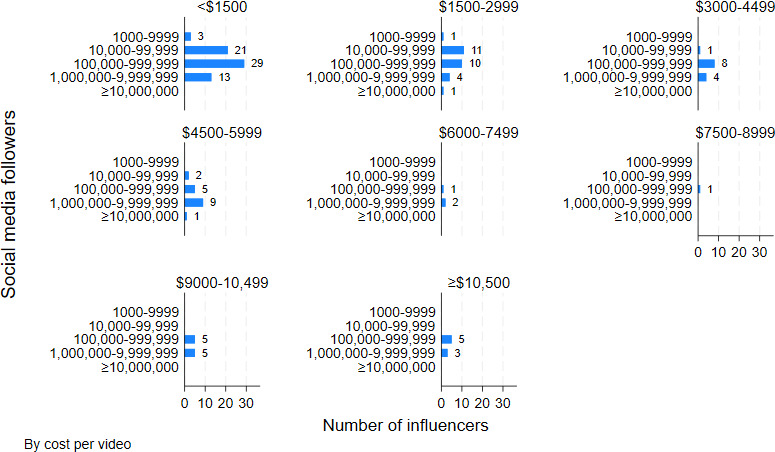
Number of influencers by social media followers and cost per video, among influencers who are popular in Atlanta, Miami, or Nashville. Influencers were identified in the Cameo Business marketplace on May 22, 2025, during the preparation of influencer-based recruitment for the GoFreshSE decentralized hypertension trial. For influencers with accounts on multiple social media platforms, we used the number of social media followers on the platform with the most followers. For example, if an influencer has 10,000 followers on TikTok and 100,000 followers on Instagram, they would be considered to have 100,000 social media followers. No influencer had fewer than 1000 followers. Data were abstracted on May 22, 2025.

We identified 147 possible influencers who were popular in Atlanta, Miami, or Nashville. Of these, 120 were popular in Atlanta, 26 were popular in Miami, and 18 were popular in Nashville. The median (IQR) cost was US $1500 (US $800 to US $5000). When comparing each influencer’s social media account with the most followers, the median (IQR) number of social media followers was 453,000 (90,100-1,100,000). Distribution of social media followers by cost per video appears in Figure 2. For instance, the highest fee was for the rapper, Juicy J, who charged US $40,000 per video and had 4.7 million followers on Facebook. The rapper Soulja Boy had the social media account with the most followers at 11.4 million on Facebook, charging US $2000 per video. The 57 potential influencers with ≥100,000 followers and a cost of less than US $3000 appear in Table 1.

After applying these major exclusions, our group opted to prioritize several factors when selecting our target influencer. First, our group had experience with running recruitment advertisements on Meta social networks and was planning on initially deploying the advertisement video on these platforms. We therefore selected persons with Facebook or Instagram accounts with a greater number of followers since we thought the influencer might be more readily recognized by users of these platforms. Second, we preferred to select a Black influencer with a greater proportion of male followers. This was because in our experience with GoFresh and GoFreshRx trials, we found it more challenging to recruit Black males than other race-sex groups. Third, the trial was open to anyone aged 18 years and older, but hypertension is more common with older ages, so we prioritized persons who were popular among older audiences. Fourth, we opted not to select influencers who were known for health-conscious messaging because we did not want to recruit a population that was more healthy than the average US adult. Fifth, we opted not to include persons whose sample videos included behaviors not in-line with AHA goals, such as smoking during the video. Finally, we opted not to select persons with noteworthy legal issues. After reviewing as a group, we selected Hue Jackson as first choice with Big Boy as alternate choice should Mr Jackson be unavailable or uninterested in completing the video.

**Table 1.** Details of 57 Cameo Business–listed influencers popular in Atlanta, Miami, or Nashville, with ≥100,000 followers on ≥1 social media platform account and <US $3000 video cost, identified during the preparation of influencer-based recruitment for the GoFreshSE decentralized hypertension trial (marketplace data abstracted May 22, 2025).

Name	Influencer specific details				Audience-specific details^a^		
	Profile tag line	Sex	Cost (USD)	Number of social media followers^a^	Most prevalent age group strata (years)	Most prevalent income group strata (USD, in thousands)	Most popular gender
Project Pat	Rapper—Three 6 Mafia	Male	$2500	IG^b^: 621,000	25-34	$60 to $80	Male (73%)
Aries Spears	Comedian	Male	$2500	FB^c^: 998,000 IG: 44,600 YT^d^: 365,000	25-34	$20 to $40	Male (84%)
Cynthia Bailey	Bravo—Real Housewives of Atlanta	Female	$2500	FB: 1,200,000 IG: 4,000,000 TW^e^: 88,200	25-34	$0 to $20	Female (77%)
Tionne “T-Boz” Watkins	Grammy-Winning Musician from TLC	Female	$2500	IG: 1,200,000 TW: 399,000 YT: 831	25-34	$0 to $20	Female (71%)
Gina Yashere	Stand Up Comedian	Female	$2400	IG: 254,000 YT: 17,000	25-34	$20 to $40	Female (73%)
Soulja Boy	Rapper	Male	$2000	FB: 11,400,000 IG: 7,300,000 TW: 5,300,000 YT: 3,400,000 TT^f^: 2,800,000	25-34	$0 to $20	Male (62%)
Henry Santos	Singer & Songwriter	Male	$2000	FB: 641,000 IG: 623,000 TW: 242,000 YT: 154,000 TT: 208,000	25-34	$40 to $60	Female (69%)
AJ McCarron	Athlete	Male	$2000	FB: 240,000 IG: 60,800	Not reported	$20 to $40	Not reported
Shawn Stockman	Member of the R&B group Boyz II Men	Male	$2000	IG: 159,000 YT: 3400	25-34	$60 to $80	Female (64%)
Steve Smith Sr	NFL Legend—Carolina Panthers | Baltimore Ravens	Male	$2000	IG: 157,000 TT: 59,100	25-34	Not reported	Male (88%)
Ozzie Guillen	MLB World Series Champ—Chicago White Sox	Male	$2000	FB: 124,000 IG: 255,000 YT: 6300	25-34	$60 to $80	Male (81%)
Marlo Hampton	Bravo—Real Housewives of Atlanta	Female	$1750	FB: 504,000 IG: 1,500,000 TW: 402,000	25-34	$0 to $20	Female (78%)
Gary Levox	Singer—Rascal Flatts	Male	$1575	IG: 214,000 TT: 909,000	18-24	$60 to $80	Female (68%)
Tituss Burgess	Actor—Singer	Male	$1500	FB: 150,000 IG: 627,000 TW: 14,200 YT: 313 TT: 46,000	25-34	$60 to $80	Female (76%)
Trina	Entertainer	Female	$1500	FB: 742,000 IG: 4,900,000 TW: 2,500,000	25-34	$0 to $20	Female (60%)
Brian & Mika Kleinschmidt	HGTV: 100 Day Dream Home/Rock the Block	Female^g^	$1499	IG: 129,000	35-44	$60 to $80	Female (85%)
Reggie Wayne	Former NFL—Indianapolis Colts	Male	$1309	IG: 106,000	25-34	$60 to $80	Male (82%)
Gizelle Bryant	Bravo—Real Housewives of Potomac	Not reported	$1250	IG: 754,000 TW: 34,600 TT: 84,400	25-34	$60 to $80	Female (86%)
Tiffany Pollard	VH1—I Love New York	Female	$1225	IG: 998,000 TW: 191,000	25-34	$0 to $20	Female (72%)
Marla Gibbs	Actress—The Jeffersons	Female	$1043	IG: 314,000	25-34	$60 to $80	Female (74%)
Michael Vick	NFL Legend—Atlanta Falcons	Male	$1000	FB: 3,000,000 IG: 1,700,000 TW: 2,500,000	25-34	$0 to $20	Male (82%)
Cassidy	(BARS) (Da Hustla) (Mr PodCass)	Female^h^	$1000	FB: 26,500 IG: 599,000 TW: 193,000 YT: 250,000	25-34	$60 to $80	Male (75%)
BRE-Z	Actress—All American, Empire	Female	$1000	IG: 1,400,000 YT: 73,400	25-34	$0 to $20	Female (74%)
Brad Guzan	MLS—Atlanta United | U.S. Men’s National Team	Male	$1000	FB: 65,000 IG: 64,600 TW: 247,000	25-34	$0 to $20	Male (75%)
LaLa Ri	RuPaul’s Drag Race	Missing	$1000	IG: 252,000 TW: 10 TT: 30,200	25-34	$60 to $80	Female (53%)
Shep Rose	Bravo—Southern Charm	Male	$1000	IG: 922,000	25-34	$20 to $40	Female (91%)
Trick Daddy	Rapper	Male	$1000	IG: 949,000	25-34	$0 to $20	Male (52%)
Fred Hammond	Gospel Icon	Male	$1000	FB: 2,400,000 IG: 930,000 TW: 497,000 YT: 3,000,000	25-34	$40 to $60	Female (59%)
Bootsy Collins	Musician—Funkadelic	Male	$1000	FB: 763,000 IG: 191,000 TW: 6200 YT: 102,000 TT: 108,000	25-34	$20 to $40	Male (66%)
Stacey Dash	Actress—Clueless	Female	$1000	IG: 128,000 TW: 474,000 TT: 294,000	25-34	Not reported	Female (55%)
Sheryl Swoopes	WNBA Legend & Hall of Famer	Female	$1000	IG: 123,000	25-34	$60 to $80	Male (50%)
Layzie Bone	Bone Thugs-N-Harmony	Male	$999	FB: 1,000,000 IG: 592,000 TW: 115,000	25-34	$60 to $80	Male (64%)
Fernando Fiore	TV Host	Male	$999	IG: 32,900 TW: 111,000	25-34	$60 to $80	Male (67%)
Jidenna	Musician	Male	$980	FB: 1,500,000 IG: 1,200,000 TW: 931,000	25-34	$0 to $20	Female (64%)
Ryan Jamaal Swain	TV/Film Actor—POSE	Male	$950	IG: 286,000	25-34	$20 to $40	Female (56%)
Isaiah Washington	Actor—Grey’s Anatomy	Male	$800	IG: 191,000	18-24	$20 to $40	Female (72%)
Big Boy	Radio Host	Male	$800	FB: 3200 IG: 589,000 TW: 210,000 TT: 775,000	25-34	$0 to $20	Male (62%)
Robyn Dixon	Bravo—Real Housewives of Potomac	Female	$750	IG: 590,000	25-34	$0 to $20	Female (87%)
KID from Kid N Play	Actor/Comedian/Artist—Kid N Play | House Party	Male	$750	FB: 130,000 IG: 265,000 TW: 49,300	25-34	$0 to $20	Male (59%)
Luenell	Actress—Comedian	Female	$700	FB: 694,000 IG: 1,100,000 YT: 769,000	25-34	$0 to $20	Female (66%)
Godfrey	Comedian	Male	$595	IG: 286,000 TW: 119,000	25-34	Not reported	Male (79%)
I’m Tyrone	Instagram Comedian	Male	$500	IG: 1,000,000 TW: 5100	25-34	$0 to $20	Male (87%)
Matt Barnes^a^	Former NBA player	Male	$500	IG: 1,000,000 TW: 518,000	Missing	Missing	Missing
Dominique Jackson	Actress & Model—Pose	Female	$500	IG: 916,000 TW: 110,000	25-34	$60 to $80	Female (56%)
Chingy	Rapper	Male	$500	IG: 255,000 TT: 98,100	25-34	$0 to $20	Female (74%)
Dana Evans	WNBA—Las Vegas Aces	Female	$500	IG: 106,000 TW: 20,100 TT: 5200	25-34	$60 to $80	Male (55%)
Flyy Soulja	Viral Star—Island Boys	Male	$500	IG: 1,400,000 TW: 167,000 YT: 315,000	18-24	$20 to $40	Male (66%)
Redman	Rapper	Male	$500	FB: 2,200,000 IG: 2,000,000 TW: 787,000 YT: 487,000	25-34	$0 to $20	Male (74%)
Gorilla Nems	Rapper—Musician—Recording Artist	Male	$500	IG: 439,000 YT: 279,000	25-34	$60 to $80	Male (84%)
Lane Kiffin^a^	Head Football Coach—University of Mississippi	Male	$350	IG: 93,400 TW: 682,000	Missing	Missing	Missing
SoYTiet	Count Master—Singer	Male	$300	IG: 1,100,000 YT: 583,000	25-34	$20 to $40	Male (65%)
Hue Jackson	Former NFL Coach—Cleveland Browns	Male	$300	IG: 2,300,000	25-34	$60 to $80	Male (74%)
Cupid	Musician—“Cupid Shuffle” “FLEX”	Male	$300	FB: 318,000 IG: 352,000 YT: 116,000	25-34	$60 to $80	Female (61%)
Safaree Samuels	VH1—Love & Hip Hop—Rapper	Male	$300	FB: 1,100,000 IG: 3,500,000 TW: 326,000 YT: 69,900	25-34	$60 to $80	Female (76%)
Ricky Williams	NFL Legend—Miami Dolphins	Male	$300	FB: 15,300 IG: 179,000 TW: 8400	25-34	$60 to $80	Male (76%)
Hassan Johnson	Actor—The Wire	Male	$250	IG: 127,000	25-34	$60 to $80	Male (60%)
J Alphonse Nicholson	Actor—P-Valley	Male	$250	IG: 687,000	25-34	$60 to $80	Female (82%)

a Several influencers paused or inactivated their accounts since the initial search, and the authors were unable to abstract these details; “missing” indicates data that were missing or could not be abstracted. The initial search was performed on May 22, 2025, and additional data were abstracted on October 20, 2025.

b IG: Instagram.

c FB: Facebook.

d YT: YouTube.

e TW: Twitter (now known as X).

f TT: TikTok.

g This is a husband and wife couple [30].

h This person is noted to be male, per their categorizations associated with their Wikipedia page [31].

### Script Development, IRB Approval, and Cameo Business Booking Request

To develop the video script used, we began by transcribing 5 sample videos created by the selected influencer from his Cameo business profile to characterize commonly used language, phrasing, and tone. Separately, a generic script was drafted by a study team member based on the design, aims, and key information from the study (Table S2 in Multimedia Appendix 1).

The generic script was then adapted using the large language model ChatGPT (GPT-4o; OpenAI), accessed through the standard ChatGPT web interface, which does not expose adjustable temperature or other decoding parameters; default settings were therefore used. Generative artificial intelligence (AI) was used solely for stylistic adaptation, matching the influencer’s tone and phrasing, and not to generate any study facts, eligibility criteria, or other substantive content, all of which originated in the study team–drafted generic script. To perform the adaptation, transcripts of 5 of the influencer’s sample videos were uploaded as examples of his typical communication style, and the model was prompted to revise the generic script to match the tone and language of those transcripts. The model’s output was then reviewed and revised by the study team for clarity, factual accuracy, and consistency with the study aims and IRB-approved content. Script development took approximately 1 week.

The final script was submitted as an amendment to the GoFreshSE study protocol to the Beth Israel Deaconess IRB. This IRB submission explicitly stated that the script was intended for use in an online video shared via social media platforms, and that there may be script adaptations. IRB review and approval took approximately 1 month. The version submitted for review was the generative AI–adapted script itself, which the IRB approved for use and adaptation.

A Cameo Business booking request specifying the goals of the video, style and tone, filming requirements, and the script was completed. This booking request is shown in Table S3 in Multimedia Appendix 1. Due to an unexpected Cameo Business script text limit of 1500 characters (including spaces), the original IRB-approved script was shortened to match the character limit.

In addition to the base video cost and its 15-day license, we opted to select a 60-day extension for a total of 75 days of use. The total booking request cost was US $660, US $300 for the video and 15-day license, US $300 for an additional 60-day license, and a US $60 service fee. The booking request was submitted on Friday, September 26, 2025. A completed video from Mr Jackson was delivered electronically 4 days later.

### Preparation of the Provided Video for Use as an Advertisement on Digital Platforms

The investigators found Mr Jackson’s video to be of very high quality as it followed the booking request instructions, including reading of the script with very little variation from what was provided. Two minor issues were identified in the provided video. First, Mr Jackson’s gaze was to the right of the camera, presumably because he was reading a printed copy of the script. Study team members thought that it would be ideal if he were maintaining eye contact with the camera. Second, Mr Jackson read very clearly, but study team members thought that the pace was slightly slower than was common in contemporary online videos. To address the eye contact issue, the NVIDIA Eyecontact model was used to adjust the gaze to the camera (NVIDIA API, NVIDIA Foundation). To address the slower pace, ffmpeg (ffmpeg team) was used to increase the speed by 5%. The speed adjustment was applied to the final edited video as the last step prior to upload. Details on both of these procedures are outlined in Table S4 in Multimedia Appendix 1. These modifications were performed under the adaptation and derivative works rights granted to licensees by the Cameo Business terms of service, which permit editing, adaptation, and the creation of derivative works provided that the watermark remains fully intact and the substantive message and any endorsement are neither altered nor misrepresented (Table 1). Our edits adjusted only the direction of gaze and the pace of delivery; they did not change the script, the claims made, or the endorsement, and the Cameo watermark was retained throughout.

As is the standard in Cameo, the video was recorded in a vertical 9:16 aspect ratio (in pixels: 720 width by 1280 height). We intended to prepare this video for distribution on Meta channels and TikTok. Meta guidelines recommend use of 1:1 (ie, square) aspect ratios to fit the many different places on their platforms on which the video may be played [32]. TikTok recommends maintaining 9:16 format [33]. As noted in Table 1, the Cameo terms do not allow removal of the watermarks, and one of the watermarks appears in the bottommost left part of the video, so it was not possible to rerender the video in an aspect ratio other than 9:16. However, the investigators discovered that the video will be vertically centered by Meta to fit 1:1 aspect ratios when appropriate. Therefore, the investigators opted to render a 9:16 aspect ratio video with the primary video content being centered in the center vertically in a 1:1 square as is shown in Figure 1.

We opted to shorten the duration of the video as an editorial decision. The entire video sped up by 5% was 1 minute and 50 seconds in length. The content was further trimmed to approximately 36 seconds in length in the main section of the video (Figure 1; see final transcript in Table S2 in Multimedia Appendix 1). The final video was rendered as a 720 × 1280 pixels MP4 file at 30 frames per second using the AVC/AAC H.264 MP4 codec. In summary, the received video was sped up by 5% (1 minute and 50 seconds in length), trimmed to an approximately 36-second main section, and combined with introduction and ending slides to yield a 44-second edited video; with the 3-second Cameo slide appended, the final deployed video was 47 seconds in length. It included an introduction slide, the main section with Mr Jackson’s Cameo video, and an ending slide as is shown in Figure 1. The introduction highlighted geographic sites of recruitment, a call to action (“Join GoFresh”), and brief study details. A brief outro listed a call to action (“Learn more and sign up...”), a domain that redirects to the study website, the funder, the IRB number, the ClinicalTrials.gov listing, and the PI name. Institutional logos from Beth Israel Deaconess Medical Center, Harvard Medical School, and the AHA were not included as they would require review by branding committees, which would introduce substantial delay in the release of the video.

Video production used Vegas Movie Studio (version 17.0; Magix Software), a proprietary nonlinear video editor. This included generation of the introduction and ending slides, trimming of the main video to length, placement of the overlay on the main section, and subtitles addition. The main section’s overlay was generated using Inkscape, an open-source vector graphics editor. A drumbeat audio sample used along with the introduction slide was generated with Drumbit, an online drum machine (João Santos). Vegas Movie Studio processed all audio by applying a noise gate filter and a compressor (1.5:1 compression starting at −24 decibels).

### Deployment on Meta Platforms

The video was uploaded to Meta’s advertising platform for display on Facebook and Instagram advertisement feeds. The video was set to target eligible ZIP codes within the study’s target region. Use of this advertising is ongoing, and details on the efficacy of this advertising modality will be evaluated in a later manuscript.

## Discussion

### Principal Findings

Here, we describe the use of Cameo Business for the rapid development and deployment of an influencer marketing video for use in clinical trial recruitment. The duration of time from script development to deployment on social media was <2 months. The total Cameo charge was US $660. We believe that these are within reach of most clinical trial’s timelines and budgets.

### Growth of Social Media Influencer Marketing in the Commercial Realm and Potential Use in Clinical Trial Recruitment

Social media use continues to climb, and most US adults have used a social media platform, including Facebook (71% have ever used), Instagram (50%), and TikTok (37%) [34]. Social media platforms continue to generate higher advertising revenues, increasing from US $41.5 billion in 2020 to US $88.7 billion in 2024 [35]. The rise of social media–based advertisement has been met with large reductions in traditional media advertisement modalities, such as print media. Traditional media now comprises only 20% of all advertisement dollars [36]. Social media influencer marketing is likewise becoming more popular and widely used by commercial brands [17]. Clinical trials have likewise begun to leverage digital tools, including social media–based recruitment, to engage study populations [37,38]. However, we are unaware of any prior descriptions of influencer marketing on social media platforms for clinical trial recruitment. We do not expect this gap to persist, as we were able to design and deploy a clinical trial social media influencer marketing campaign with relative ease.

### Our Experience in Developing and Releasing a Social Media Influencer Marketing Campaign for Clinical Trial Recruitment

We were surprised by the rapidity with which we were able to move this pilot forward. Our specific scenario benefitted from several institutional policies and expertise from the study team that may limit other teams’ progress. First, some IRBs might decline to review draft scripts and instead may permit only review of a final video. By reviewing the scripts prior to their use in the recording process, we were able to ensure that the narrative content of the video was okay to use prior to video recording. IRBs that review only the final video may disapprove the video based upon script issues. Such corrections at this stage may be addressed only with rerecording of the video, which would bring substantial costs and delays. Second, the PI’s institution did not require institutional-level review of the video because it did not include any institutional branding. Other institutions may have policies requiring institutional-level review of any video produced, regardless of branding use. Third, our study team had expertise in digital trial recruitment and video production. Other groups may need to hire consultants with website design experience, social media advertising experience, or video production experience to complete such a project.

### Unanticipated and Anticipated Challenges in the Development and Release of a Social Media Influencer Marketing Campaign

There were 2 unforeseen challenges in our pilot. First, while the Cameo Business website allows for rapid selection of influencers, it is limited in its guidance of what is required to complete the booking request since the booking request cannot be seen until the actual order is generated. We were specifically surprised that the script was limited to 1500 characters (including spaces) for our script and had intended to upload a script that was 2627 characters (including spaces) in length. This discovery led to a last-minute script revision. In retrospect, a lengthier script is not particularly compatible with conventional video-based recruitment, and the transcript of the final video was only 543 characters (including spaces). In future videos, we will prepare a much shorter script than originally done in this pilot. Second, we were surprised that Meta required videos to render in a 1:1 square since not all of the placements for video advertisements would support a 9:16 aspect ratio. This discovery required minor reworking of the text and image framing. Future videos will account for framing in a 1:1 vertically centered box from the onset.

There were several anticipated issues that we were able to mitigate and also potential issues that did not arise and were avoided in retrospect. First, we were concerned about the technical quality and visual framing of the video. In the booking request, we made specific requirements for adequate front lighting, no distracting background lighting, and a clean camera lens. Although the descriptions given were brief, the selected influencer (Mr Jackson) adhered to these instructions closely, thus providing a high-quality video.

We later realized that we had not provided specific instructions regarding video framing; however, the influencer was well-positioned within the frame of the video and was particularly appropriately centered for the 1:1 vertically centered square framing. Had he been positioned substantially closer to the camera, it would have been challenging to fit him in the frame alongside the added text and subtitles. Based on this experience, future requests will include clear instructions on framing and maintaining an appropriate distance from the camera. Finally, although we did not ask him to speak slowly and clearly, he did so unprompted. Future video requests should be clear about speaking slowly and clearly to ensure ease of comprehension.

### Next Steps in Our Social Media Influencer Marketing Campaign

The current plan for this video is to show it as a paid advertisement in ZIP codes from which the trial is recruiting, which is similar to our prior strategies that used still recruitment images on Meta platforms. An alternative social media influencer marketing approach is to partner with an influencer who would share recruitment materials from their personal social media accounts to their followers. While this approach might work for certain trials that are recruiting across broad geographic areas with high recruitment numbers, that approach would not work for GoFreshSE since it is a relatively small trial that is recruiting from only 3 geographic areas in the United States. Importantly, the influencer we chose (Mr Jackson) has 2.3 million followers on Instagram, and blasting our recruitment video to such a large audience all at once would cause major issues with the stability of the recruitment website and also the ability to rapidly screen potentially eligible participants within the study’s short time frame. Study teams that are considering advertising in which an influencer distributes recruitment materials to millions of followers will need to navigate several additional problems that we did not run into. First, there is no mechanism from Cameo Business to hire an influencer to share your advertising materials on their personal accounts. Study teams would need to directly inquire with influencers on whether they would be willing to share materials and establish such a cost before hiring them on Cameo Business to make their video in the first place. For musicians, actors, and athletes, this would probably need to occur through their agents. For other people famous from nontraditional means (eg, someone who simply grew a large following through social media engagement), there may not be a clear means to establish such an arrangement. Second, there will need to be substantial technical support to ensure that the recruitment website will not crash from an immense number of visitors arriving in a short amount of time. This may require high-cost consultants and server fees. Third, there will need to be efficient mechanisms to determine the eligibility of possible participants before they interact with the study team.

### Principles and Trade-Offs for Selecting an Influencer

Selecting an influencer requires balancing reach, audience alignment, and cost. Several established communication concepts can inform these choices [39-42]. Source credibility theory holds that a communicator’s perceived expertise, trustworthiness, and attractiveness shape the persuasiveness of a message. Homophily, the tendency for people to be influenced more by others who resemble them, and parasocial interaction, the one-sided sense of familiarity that audiences develop with media figures, help explain why an influencer whose audience is demographically similar to the target population may engage that population more effectively than a generic advertisement. These constructs informed our prioritization. Because we previously found Black men more difficult to recruit than other race-sex groups, we prioritized a Black influencer whose audience skewed male; evidence that racially concordant messengers increase engagement with preventive cardiovascular care among Black men supports this choice [43]. We also weighed cost against reach. Within our qualifying pool, price and follower count were only loosely related, and several influencers with very large followings charged modestly. Ultimately, audience fit and demographic alignment, rather than price, drove our final selection. We emphasize that our thresholds (≥100,000 followers and <US $3000 per video) were pragmatic starting points rather than empirically validated selections; we did not test alternative cutoffs, and other teams should adapt these constraints to their own reach requirements, budgets, and target populations.

### Authenticity and Generative AI Governance in Influencer-Based Recruitment

Editing an influencer’s delivered video raises authenticity questions that warrant explicit attention in a recruitment context, where participant trust is paramount. We distinguish presentation-level modifications (eg, redirecting gaze to the camera or modestly adjusting pace) from substantive modifications that would change the words spoken, the claims made, or the endorsement given. Our edits were confined to the former and preserved the influencer’s message in full, consistent with the Cameo Business terms, which permit adaptation and derivative works but prohibit altering or misrepresenting the substantive message and require the watermark to remain intact. We report the specific tools and parameters used for every modification so that the nature and magnitude of each edit are transparent and auditable. More aggressive synthetic alteration (eg, using generative video tools to change spoken content or fabricate statements the influencer did not make) would raise materially different authenticity and ethical concerns and could breach licensing terms; investigators should avoid such modifications and, where any edit could plausibly affect approved messaging, confirm the scope of IRB approval.

Our use of a large language model illustrates a governance approach that other investigators may adapt. We restricted the model’s role to stylistic adaptation rather than content generation, so that no study claim, eligibility criterion, or other substantive message originated with the model; the factual content was human-authored, and the model altered only voice and tone. Two independent layers of human oversight followed: First, the study team reviewed the model’s output, and second, an IRB reviewed and approved the resulting script before deployment. Because the adapted script, and not a generic placeholder, was the version the IRB reviewed and approved, the messaging delivered to potential participants had been vetted in the exact form derived for use, mitigating concerns that model-introduced phrasing could drift from approved content or overstate study benefits. We recommend that investigators explicitly disclose generative AI involvement to their ethics board, both to support transparent oversight and because reporting expectations around AI use in research communications are evolving [44,45].

### Ethical Considerations and Participant Protection

Beyond standard advertisement review, influencer-based recruitment introduces distinct ethical considerations. A recognizable public figure may be perceived as personally endorsing the intervention, which could exert undue influence or blur the line between advertising and an invitation to research [46], particularly among audiences with limited research literacy. A related risk is the therapeutic misconception [47], in which prospective participants assume that study participation is designed primarily to benefit them rather than to answer a research question. We took several steps to mitigate these risks. The video explicitly described the study as a clinical trial with random assignment (framed as a “coin toss”) to either home-delivered groceries or a grocery stipend, avoiding any implication of guaranteed personal benefit, and it directed viewers to a study website that presents full eligibility criteria, the funder, the IRB number, ClinicalTrials.gov registration, and study contact information. The influencer described the trial and its randomization rather than personally vouching for the intervention’s efficacy. We also note that the promotional phrasing in our booking request (eg, describing the influencer as “the first famous person to help recruit for a clinical trial,” Table S3 in Multimedia Appendix 1) was directed to the influencer to encourage participation and did not appear in participant-facing materials. Investigators using this modality should similarly ensure that enthusiasm in recruitment messaging does not overstate benefit or obscure the research nature of the study.

### Equity, the Digital Divide, and Decentralized Trials

As a decentralized trial that delivers groceries and sends examiners to participants’ homes, GoFreshSE intentionally serves communities across the Southeastern United States, which makes the equity implications of digital recruitment especially salient [48]. Influencer videos distributed as paid social media advertisements reach only those people who are online and are subject to platform ad-delivery algorithms, which can unevenly expose demographic groups [49]. Cameo Business permits filtering an influencer’s audience by income, and Meta permits granular advertisement targeting; either could systematically over- or underreach particular income or demographic strata. To avoid narrowing reach by socioeconomic status, we did not apply audience income filters when selecting an influencer, and we targeted advertisements by the ZIP codes from which the trial recruits rather than by income. Because paid social media advertising inherently excludes those without reliable internet access [34,50], we used it as one component of a multimodal strategy that also includes patient portal messaging and community-based recruitment. Study teams adopting influencer-based recruitment for populations affected by the digital divide should be deliberate about audience and targeting filters, monitor the demographic characteristics of those who respond, and pair digital outreach with nondigital channels to avoid compounding existing disparities in research participation [11,51].

### Limitations

This tutorial has limitations. First, it describes a single implementation, and its generalizability depends on conditions that may not hold for every team: Cameo Business must offer influencers whose audiences match the target geography, those influencers must be willing to record health-related content, and the chosen platforms must permit the intended advertising. Second, we deliberately did not evaluate the effectiveness of this modality. Because recruitment is ongoing and a credible assessment requires the full recruitment period and an appropriate comparator, we do not report click-through rates, cost per screened or enrolled participant, or comparative yield against other channels; these are the planned end points of a separate analysis that will also compare an influencer-delivered video with a study team–delivered video. Readers should therefore regard the present work as establishing feasibility and a reproducible procedure rather than demonstrating comparative effectiveness. Third, the Cameo Business marketplace and its pricing change over time, and our influencer pool was abstracted in 2025. The specific influencers and fees we report may not remain static over time, although the workflow is. Finally, the audience attributes provided by Cameo Business are platform-derived estimates that we could not independently verify.

### Conclusions

Herein, we present one study’s approach for the development and deployment of a social media influencer marketing video. To our knowledge, this is the first description of influencer marketing as a clinical trial recruitment modality and the first tutorial to document the procedure end to end from marketplace selection and ethics review through video postproduction and deployment. It differs from existing tutorials on digital trial recruitment, which have focused on conventional social media advertisements, patient portal messaging, or recruitment websites, by addressing the distinct contractual, technical, and ethical steps that influencer-based recruitment entails. For investigators, it offers a low-cost, rapidly executable approach to producing recruitment videos featuring recognizable figures, together with guidance on the generative AI, authenticity, and equity considerations that accompany it. Persons interested in approaching this work should review Table S1 in Multimedia Appendix 1. Future work will describe the efficacy of this modality in comparison with other advertising modalities and determine whether a recruitment video with an influencer outperforms a recruitment video with a noninfluencer, study team member.

## Supplementary material

10.2196/92813 Multimedia Appendix 1 Supplemental tables.

## Abbreviations

AHA: American Heart Association
DASH: Dietary Approaches to Stop Hypertension
GoFresh: Groceries for Black Residents of Boston to Stop Hypertension Among Adults Without Treated Hypertension
GoFreshRx: Groceries for Black Residents of Boston to Stop Hypertension Among Adults With Treated Hypertension
GoFreshSE: Groceries for Residents of Southeastern USA to Stop Hypertension
IRB: institutional review board
PI: principal investigator
