# Supplementary material for: Social Media Influencer Marketing as a Clinical Trial Recruitment Modality: Tutorial Informed by One Study’s Approach
Source: J Med Internet Res. 2026 Aug 11;28:e92813. doi: 10.2196/92813 (PMC13482797; doi:10.2196/92813)
Supplement: Supplementary file 1 [file jmir-v28-e92813-s001.pdf]

This document includes supplemental tables to be included with the manuscript "***Social media influencer marketing as a clinical trial recruitment modality: A tutorial informed by one study's approach***" (https://www.doi.org/10.2196/92813).

Table S1 – Preparation framework for using Cameo Business to develop a social media influencer marketing campaign. This checklist generalizes the procedure used for the GoFreshSE decentralized hypertension trial (described in the main text) into a reusable, step-by-step guide that other study teams can adapt for their own recruitment campaigns; the thresholds and options shown are suggested starting points rather than requirements.

**Preparing a recruitment website for incoming traffic**		
**Purchase a domain that leads to your website.** Suggest using instantdomainsearch.com to find an available domain and using hover.com to purchase that domain. Suggest purchasing a domain that ends in ".com", is simple to remember, and is descriptive.	☐ Done	
**Develop a recruitment website.** Suggest using a commercial website building platform (e.g., Weebly, Squarespace, Hostinger). Have this IRB approved. Consider using the privacy-oriented website analytics platform, Matomo, to monitor visitation.	☐ Developed<br>☐ IRB approved<br>☐ Deployed	
**Embed a secure signup form on your website.** REDCap or Qualtrics forms linking to a secure research database can be embedded on your recruitment website with an iframe. Example coding can be found here https://ways2web.weebly.com/h-iframe.html. Consider including questions about how the person heard about the study (e.g., "Video on Facebook or Instagram", "A friend", "A printed Flyer"), basic demographics, and eligibility criteria.	☐ Developed<br>☐ IRB approved<br>☐ Deployed	
**Develop staff protocols for monitoring and following up on signups.**	☐ Done	
**Preparing for social media advertisement deployment**		
**Setting up social media marketing tools.** Common platforms include Meta (Facebook & Instagram), TikTok, and YouTube. Pew Research Center provides data on which are most popular overall and by demographic group. https://www.pewresearch.org/internet/fact-sheet/social-media/?utm_source=prcsite&utm_medium=topic-summary&utm_campaign=social-media	☐ Select your target social media platform(s)<br>☐ IRB approval to use that platform for recruitment purposes.<br>☐ IRB approval to use the script that will be used in the video. If possible, get approval for the script and derivations of that script, including videos.<br>☐ Identify geographic and sociodemographic targets for your eventual ad campaign.<br>☐ Determine how payments for ad campaigns will occur (e.g., on an institutional credit card?)<br>☐ Develop a protocol for running the social media campaign, including monitoring costs and effectiveness.<br>☐ If your website allows, set up tracking URLs to use in your campaign to track flow to your website.	
**Optimizing your Cameo Business Marketplace search (https://www.cameo.com/business/browse)**		
**Talent-specific queries**		
**What category of influencer do you prefer?** We suggest not limiting to a specific category or subset of categories unless you have a strong rationale to do so.	☐ Doesn't matter<br>☐ Actors<br>☐ Athletes<br>☐ Comedians<br>☐ Creators	☐ International<br>☐ Musicians<br>☐ Professionals<br>☐ Reality TV<br>☐ Sports commentators
**What is your estimated budget available to pay to Cameo?** The price shown on the Cameo marketplace only allows 15 days of use. Extending the license to 180 days will about triple the price.	Estimated budget: _____<br><br>Target price = estimated budget/3 = _______ or less	

**What is the minimum number of followers that you want your influencer to have?** We suggest >100,000 in ≥1 social media account.	☐ Doesn't matter<br>☐ ≥100,000	☐ ≥250,000<br>☐ ≥500,000<br>☐ ≥1,000,000
**What sex do you prefer for an influencer?** We suggest not limiting to a single sex unless you have a strong rationale to do so.	☐ Doesn't matter<br>☐ Female<br>☐ Male	
**Audience-specific queries**		
**Do you want the influencer to have a substantial number of followers that are of a specific sex?** Defined as ≥40% of their followers being of that sex. We suggest not limiting to a specific sex unless there is a strong rationale to do so.	☐ Doesn't matter<br>☐ Female<br>☐ Male	
**Do you want the influencer to have a substantial number of followers that are of a specific age band?** Defined as ≥20% of their followers being of that age band. The minimum age is 18 and maximum age is 54. We suggest not limiting to a specific age band or subset of age bands unless there is a strong rationale to do so.	☐ Doesn't matter<br>☐ ≥18 to 24<br>☐ ≥25 to 34<br>☐ ≥35 to 44<br>☐ ≥45 to 54	
**Do you want the influencer to have a substantial number of followers that are from a specific country?** Defined as ≥5% of their followers being from that country.	☐ Doesn't matter<br>☐ Yes: ______	
**Do you want the influencer to have a substantial number of followers that are from a specific city?** Defined as ≥1% of their followers being from that city.	☐ Doesn't matter<br>☐ Yes: ______	
**Do you want the influencer to have a substantial number of followers that are within certain income ranges (USD)?** Defined as ≥20% of their followers being in that range. We suggest not limiting to a specific income range unless you have a strong rationale to do so.	☐ Doesn't matter<br>☐ $0 to $20k<br>☐ >$20 to $40k<br>☐ >$40 to $60k<br>☐ >$60 to $80k<br>☐ >$80K	
**Vetting your influencer(s)**		
**Your selected influencer's personal brand and legacy will reflect on your study, institution, and funding organization**. Ensure that there isn't undue notoriety for your selected influencer(s).	Suggest the following steps:<br>☐ Review online biographies on the influencer(s), e.g., Wikipedia.<br>☐ Identify colleagues, family members, and/or friends who follow the influencer(s) and/or the profession for which they are known and ask them their opinion of the influencer(s) and whether they'd be appropriate to use for recruitment to your study.<br>☐ Review a sample of online postings from your influencer(s). Consider the activities and themes of these postings and whether they are consistent with your research aims. E.g., an influencer smokes in their videos, they may not be an ideal influencer to use in a cardiovascular prevention-focused trial.	
**Preparing your Cameo Business booking request and Cameo character limit (character count includes spaces)**		
**Prompt 1: What's the goal of this video? (≤500 characters).** The influencer has to accept the offer to make the video. We think it is important to write	Consider including the following:<br>☐ Who you are<br>☐ The goals of the trial<br>☐ That the video will be used to recruit to the trial	

this and other sections in a warm and encouraging tone.	☐ Where the video will be shown<br>☐ Who is sponsoring the trial.
**Prompt 2: Tone and style (≤200 characters)**	Suggest asking the person to use their usual tone. The style will be dictated by the script that is developed and may not to be commented on if the style is clear from the script itself.
**Prompt 3: Filming requirements (≤200 characters)** If running over in length, some of the details here can be included in prompt 2 or prompt 4.	Set-up options:<br>☐ Minimize light coming from behind<br>☐ Ensure adequate front-lighting, either natural light (e.g., sitting in front of a window) or external lighting (e.g. a ring light)<br>☐ Ensure a clean camera lens, use a dry cloth, paper towel, or tissue to clean the lens before recording<br>☐ Consider specifying what is in the background<br>☐ Consider specifying outfits<br><br>Recording options:<br>☐ Self-held selfie video or<br>☐ Phone in a stand (specify how far away it should be placed or how the person should be framed, such as in a box, vertically centered in the video)<br><br>☐ Vertical filming like on TikTok or<br>☐ Wide-screen filming like a movie<br><br>Specifics about reading the script:<br>☐ Read the script, adding any riffs or improvs as you see fit or<br>☐ Read the script without adding additional content<br><br>☐ Read script in your usual speaking speed or<br>☐ Read the script slightly slower than your usual speaking speed
**Prompt 4: Things to avoid (≤300 characters)**	☐ If the trial relates to a sensitive topic or marginalized group, consider giving anticipatory guidance on the topic or group as it relates to the influencer's role.
**Prompt 5: Other things to know (≤250 characters)**	☐ Consider thanking the influencer for their participation.
**Prompt 6: Exact script (≤1500 characters)** There is also an option to include talking points and allow the person to ad-lib from those points. We suggest using the exact script option.	Full script goes here. We suggest getting IRB approval of the script before submitting the Cameo Business booking request.
**Prompt 7: Additional notes (≤150 characters)**	☐ Spell out pronunciations of words they may not know how to pronounce here.
**Preparing the video**	
**Video editing considerations.** Cameo provides up to 60 days for editing the video before the license begins. The license may be activated early if the full 60 days are not needed.	☐ Does it require re-approval from the IRB?<br>☐ Add the name of the influencer and reason for fame as overlaying text<br>☐ Add recruitment website domain name as overlaying text<br>☐ Add subtitles

	☐ Center text and influencer in 1:1 vertically-centered box<br>☐ Add end frame with IRB number, funder, PI name, and ClinicalTrials.gov number<br>☐ Recommend ≤60 seconds in length<br>☐ Consider not including unnecessary logos (e.g., institutional logo or funder logo) that may require extra review and approval from brand committees.

Table S2 - Development of the script and final transcription from the video

<table>
<tr><td>

Original draft of a generic script written by the study team. This was written in a generic tone that might be used by any influencer. This was IRB approved for use and adaptation.

Hey, friends—[Name] here, coming to you live.
We all know that science is awesome. But did you know that you can be part of scientific discovery by EATING FOOD.
So, here's the scoop. The American Heart Association does great things like teaching people how to do CPR. But that's not all -- the American Heart Association also supports scientists who are doing cutting-edge heart health research. Diet plays a big role in health and scientists sponsored by the American Heart Association are testing how diet relates to blood pressure.

They want to see if home-delivered, heart-healthy foods can actually lower blood pressure. No, this isn't another one of those "try my new kale smoothie" infomercials. It's real science, with real food, and you could be part of it. The scientists running this clinical trial are looking for 150 volunteers, at least 18 or older, who either have elevated blood pressure or are already on treatment for high blood pressure. And not just anywhere—this is a Southern road trip: I'm talking Atlanta, Nashville, and three Florida hotspots: Gainesville, Jacksonville, and Miami. If you live in one of those cities, congratulations: you've already passed the first test.
Now, here's where you get to play the dietary lottery. The scientists will basically flip a coin to see what diet intervention you'll get.
Heads: They'll get heart-healthy meals delivered to their door. Picture this: lean proteins, whole grains, veggies so crisp they'll make your kitchen jealous. Ordering is all online along with some help from a registered dietitian, and boom! Meals delivered to the doorstep.
Tails: That's the other half of people in the trial. They'll get a $300 grocery stipend to unleash their inner food shopper. Who doesn't like someone else paying part of your grocery bill?

But wait—there's more science! (I know, I sound like a late-night infomercial, but stick with me.) At the start and end of the trial, a trained medical examiner will swing by your house. They'll measure your blood pressure and draw some blood for lab magic. Don't worry, they're friendly people.
In between, you'll use a home blood-pressure monitor—think of it as your personal health sidekick. It'll record your readings throughout the study, sending everything back to the research team.

So why do it? Besides bragging rights—"Oh, you binge-watched Netflix? I participated in a randomized controlled trial."—you'll help answer a big question: can getting healthy food mailed to you actually change your blood pressure trajectory? Plus, you might learn a thing or two about what to eat along the way.
If you're in Atlanta, Nashville, Gainesville, Jacksonville, or Miami, and you're ready to have your heart and taste buds team up, head to [final URL here]. Sign up, chow down, and help us prove that convenience and cardiovascular health can ride shotgun together.

</td></tr>
<tr><td>

ChatGPT 4o adapted script to match transcribed videos from Hue Jackson. This was IRB approved for use and adaptation.

Hey hey, how y'all doing? This is Hue Jackson, coming to you live—and listen, I've been a part of some big playbooks in my day, but this right here? This might be one of the most important ones.

So here's the deal. The American Heart Association—yeah, those folks who teach CPR and help save lives—they're also doing something else. They're backing some smart, dedicated scientists to answer a big question:
Can eating the right food, delivered right to your door, actually help lower blood pressure?
And let me tell you—it's not some health nut nonsense. This is real food, based on the DASH eating plan—that stands for Dietary Approaches to Stop Hypertension. That's lean meats, whole grains, low-fat dairy, fruits, and vegetables. Not kale powders. Not miracle juices. Just solid, balanced groceries.

Now you might be wondering—how do I get in on this?

</td></tr>
</table>

If you’re 18 or older, have high blood pressure or are being treated for it, and live in one of these places: Atlanta, Nashville, and Florida outside of the panhandle – then guess what? You’re already on the field.
Here’s how it works: This is a clinical trial, and there are two groups. It’s basically a coin toss. Heads, you get dietitian-assisted groceries that match the DASH plan, delivered right to your home for free. That’s right—you’ll get connected online or by phone with a registered dietitian who helps you order food through a virtual supermarket. No guesswork. No hassle.
Tails, you get a $300 grocery stipend. You do the shopping, your way, with the cash to back it up. Either way? You win.

But listen—this is a commitment. It’s a 4-week program. Before and after, a trained technician will come to your home to check your blood pressure, your weight, and do a little lab work. You’ll also complete some surveys and provide your medical history.
And during the study, you’ll take your blood pressure at home three times a day for 5 days using the monitor they give you—no need to write anything down. The results upload automatically to their system. That’s it.

So why do it?
Because this ain’t just about you—it’s about science. It’s about helping answer a real question: can we use smart grocery access to fight high blood pressure and heart disease?
And come on—how many people get to say they helped advance medical research just by eating good food?

So visit GO FRESH STUDY DOT COM, that’s GO FRESH STUDY DOT COM, and sign up. If you’re in Atlanta, Nashville, Florida outside of the panhandle, you’re already in the red zone. All you gotta do now is finish the drive.
Help science. Eat well. Lower that blood pressure.
Time to get right. Bye for now.

Final script. The ChatGPT 4o script uploaded to Cameo Business in the booking request. It was abbreviated to be <1,500 characters. This was read nearly verbatim by Mr. Jackson.

The American Heart Association is backing some heart disease researchers who are trying to answer a big question: Can eating the right food, delivered to your door, actually help lower blood pressure?

This is real food, based on the DASH eating plan. Lean meats, whole grains, low-fat dairy, fruits, and vegetables. Solid, balanced groceries.

Here’s how this Go Fresh study works. It’s basically a coin toss.

Heads: a dietician helps you select food from an online store that matches the DASH plan. This food is delivered right to your home for free. No guesswork. No hassle.

Tails: you get a $300 grocery stipend. You do the shopping, your way, with the cash to back it up.

Either way? You win.

This is a 4-week commitment. Before and after, a technician will come to your home to check your blood pressure, your weight, and do a little lab work. You’ll fill out some surveys and provide some medical history too. They’ll also give you a monitor to track your blood pressure at home. That’s it.

If you’re 18 or older, have high blood pressure or are on meds to lower blood pressure, and live in Atlanta, Nashville, or Florida outside the panhandle – then you’re already on the field.

So why do it?

Because this ain't just about you. It's about helping answer a real question: can we use smart grocery access to lower blood pressure and fight heart disease?<br><br>And come on—how many people get to say they helped out just by eating good food?<br><br>Visit GO FRESH STUDY DOT COM and sign up.
**Final video transcript** after editing was applied.<br><br>Can eating the right food delivered to your door actually help lower blood pressure?<br><br>Here's how this GoFresh study works. It's basically a coin toss.<br><br>Heads: a dietitian helps you select food from an online store that matches the DASH plan. This food is delivered right to your home for free.<br><br>Tails: you get a $300 grocery stipend<br><br>Before and after, a technician will come to your home to check your blood pressure, your weight, and do a little lab work.<br><br>If you're 18 or older and have high blood pressure, visit gofreshstudy.com and sign up today.

Table S3 - Cameo Business booking request

Cameo Business prompt: What's the goal of this video? We're researchers at the Beth Israel Deaconess Hospital in Boston and the University of Vermont (UVM) who are recruiting for a clinical trial called GoFresh Southeast. People living in Georgia, Florida, or Tennessee with high blood pressure will be randomized to healthy DASH diet foods in the mail or a stipend for groceries. It tests if diets can lower blood pressure. The trial is sponsored by the American Heart Association. We'll use the video on Facebook/Instagram, etc. for trial recruitment.
Cameo Business prompt: Style and tone Your usual charming self!
Cameo Business prompt: Filming requirements A selfie video please! We're providing a script for you to read (please read it in entirety), but please feel free to add whatever riffs you'd like while reading it in addition to what is provided.
Cameo Business prompt: Things to avoid Please avoid bright lights behind you. It'd be great if you had some nice natural light or a ring light in front of you to help with video quality. Please use Kleenex to clean your front-facing camera before recording.
Cameo Business prompt: Other things to know You're the first famous person to help recruit for a clinical trial! It's for a great cause. We're hoping to discover ways to help lower blood pressure and lower risk of heart disease. Thx! Tim (Tim Plante MD) [Redacted contact information]
Cameo Business prompt: Exact script [The final script from **Supplemental Table 2** was pasted here.]
Cameo Business prompt: Additional notes Feel free to riff in addition to the script above. DASH is pronounced just like you think, "dash". Thanks so much!

Table S4 - Correcting eye contact and adjusting speed

Correcting eye contact with NVIDIA Eyecontact (https://build.nvidia.com/nvidia/eyecontact), which assumes that (1) WSL is installed on a Windows PC and (2) that the raw video is available as “cameonoeyefix.mp4” in the home directory.

- Step 1: Windows Subdistribution for Linux (WSL) 2.3.26 was installed on a Windows 11 computer. This included Ubuntu 24.04.2 LTS as the Linux distribution.
- Step 2: The rendered video (“cameonoeyefix.mp4”) was copied/pasted into the home directory for the Ubuntu system using Windows Explorer:
    - \\wsl.localhost\Ubuntu-24.04\home\[username]\
- Step 3: An account and API key were obtained at https://build.nvidia.com/nvidia/eyecontact
- Step 4: Git and Python were installed locally. The provided code on the API code page (https://build.nvidia.com/nvidia/eyecontact/api) was modified to run python from a virtual environment as WSL limits ability for PIP package installation in the default installation of Python 3. Example commands follow. Please note that for the final line, the function-id and API key will need to be obtained from the API code page on NVIDIA’s website.
    - sudo apt-get update
    - sudo apt-get install git
    - sudo apt-get install -y python3 python3-pip python3-venv
    - python3 -mvenv /var/tmp/v-3.12
    - . /var/tmp/v-3.12/bin/activate
    - git clone https://github.com/NVIDIA-Maxine/nim-clients.git
    - cd nim-clients/eye-contact/
    - pip install -r requirements.txt
    - cd scripts
    - python eye-contact.py --preview-mode \
      --target grpc.nvcf.nvidia.com:443 \
      --function-id [function-id here] \
      --api-key [API key here] \
      --input ~/cameonoeyefix.mp4 \
      --output ~/cameowitheyefix.mp4
- Step 5: The “cameowitheyefix.mp4” file was copied from the Ubuntu home directory and pasted in the local research folder on Windows.

Adjusting video speed with ffmpeg version 6.1.1. This assumes that (1) WSL is installed and (2) that the raw video is available as “cameovideonormal.mp4” in the home directory as noted in steps 1 and 2 above

- Step 1: ffmpeg was installed on Ubuntu on with the following commands:
    - sudo apt-get update
    - sudo apt-get install ffmpeg
- Step 2: ffmpeg was used to increase the speed of “cameovideonormal.mp4” by 5% and save the sped up version as “cameovideofast.mp4” with the following command:
    - ffmpeg -i cameovideonormal.mp4 -filter_complex "[0:v]setpts=0.9524*PTS[v];[0:a]atempo=1.05[a]" -map "[v]" -map "[a]" cameovideofast.mp4
    - Components of this command:
        - -i cameovideonormal.mp4
            - Name of the input file. The full directory did not need to be included as the video was saved in the home directory of the Ubuntu system.
        - -filter_complex "[0:v]setpts=0.9524*PTS[v];[0:a]atempo=1.05[a]"
            - Applies a filter to the video stream’s presentation timestamps (PTS) to make it play 5% faster. The conversion is computed as 1/1.05 = 0.9524*PTS. The resultant video is named “v”.
            - Applies a filter to the audio stream that is 5% faster. This conversion is computed as atempo=1.05. The resultant audio is named “a”.
        - -map “[v]” -map “[a]” cameovideofast.mp4
            - Include the resultant audio in the output file named “cameovideofast.mp4”, which is to be saved in the home directory of the Ubuntu system.
- Step 5: The “cameovideofast.mp4” video was copied from the Ubuntu home directory and pasted in the local research folder on Windows.
